# Electrical muscle stimulation towards self-physiotherapy on myofascial pain syndrome

**DOI:** 10.3389/fresc.2026.1817369

**Published:** 2026-05-14

**Authors:** Seekaow Churproong, Benjamin Metcalfe, Polly Mcguigan, Dingguo Zhang

**Affiliations:** 1 Department of Electronic and Electrical Engineering, University of Bath, Bath, United Kingdom; 2 Institute of Medicine, Suranaree University of Technology, Nakhon Ratchasima, Thailand; 3 Department of Health, University of Bath, Bath, United Kingdom

**Keywords:** electrical muscle stimulation, myofascial pain syndrome, stretching, surface electromyography, ultrasound imaging

## Abstract

**Background:**

Myofascial trigger points (MTrPs) are the cause of pain from myofascial pain syndrome (MPS), frequently in the trapezius muscle (TM). Working adults often avoid treatment due to the need for therapist assistance and limited time.

**Methods:**

This study aimed to investigate whether electrical muscle stimulation (EMS) and active stretching (AS) can be used as a method for the self-physiotherapy of MTrPs in the TM, and to evaluate the effectiveness of the EMS + AS technique, which resembles a therapist's resistance in passive stretching (PS). Two experimental studies were conducted: Experiment 1 (8 adults with MPS) assessed TM changes via ultrasound imaging, and Experiment 2 (65 adults with MPS) examined changes in pain intensity (PI), pressure pain threshold (PPT), and surface electromyography (sEMG) activity, along with participants’ feedback. A one-minute treatment duration was delivered for each of condition in random order.

**Results:**

Experiment 1 suggests a possible trend that EMS + AS produces consistent reductions in TM thickness over 10 s; however, no significant differences were observed between time points or treatments (*p* > 0.05). Experiment 2 showed that EMS + AS significantly reduced PI compared with PS (17.50% difference, *p* = 0.047). Participant feedback showed a significant preference for EMS + AS over PS (51.40% difference, *p* = 0.002). Participants with prior stretching experience were 5.11 times more prone to correctly identify both exercise types as beneficial (OR = 5.11, *p* = 0.014).

**Conclusion:**

EMS + AS is a practical self-physiotherapy approach that does not require therapist intervention, as objective physiological outcomes did not differ significantly between therapies and the method can be conveniently performed in office or home settings.

## 1 Introduction

Myofascial pain syndrome (MPS) is diagnosed by the finding of myofascial trigger points (MTrPs), and is highly prevalent in the neck and upper back (1). The upper and middle fibers of the trapezius muscle (TM) are most frequently affected by MTrPs (2). MTrPs are hypersensitive, palpable nodules in a taut muscle band that irritate local nociceptive receptors. In pain clinics, up to 80% of patients present with MPS, characterized by either active MTrPs (spontaneous pain) or latent MTrPs (local tenderness upon pressure) (1, 3). Although the pathogenesis of MTrPs is not yet fully understood (4), MTrPs cause increased pain intensity (PI) and decreased pressure pain threshold (PPT). The severity of pain significantly limits work function (5), which is associated with acute changes in surface electromyography (sEMG) activity during TM function (6).

A literature review of current therapies for MPS, as indicated by Guzmán Pavón et al. (2022), shows that manual therapies, including stretching and combination with other interventions, are non-invasive and can provide medium reduction in PI and improvement in PPT, compared with control groups (7). However, a significant portion of working adults avoid treatment due to several barriers including the need therapies assistance, clinical time is limited, transportation challenges, distance to specialists, and a lack of understanding or distrust in treatments (8). Therefore, self-physiotherapy is important for MPS rehabilitation. Muscle stretching is the first line of physiotherapy available to reduce muscle tension and enhance blood circulation (9). Self-stretching or active stretching (AS) continues to be a simple way to relieve muscle tightness. Multiple studies indicate that both AS and passive stretching (PS) by a therapist can be effective in improving muscle flexibility and reducing pain associated with MTrPs (10). However, AS offers greater convenience as it can be performed independently and can be combined with other MPS therapies, unlike PS, which requires external assistance and may not be feasible for individuals alone or without access to a therapist. This limitation highlights a gap in care, as no effective self-physiotherapy for MPS stretching is currently available. To address this, a develop an effective self-physiotherapy by applying electrical muscle stimulation (EMS) in combination with AS is proposed as a practical, accessible, and efficient self-physiotherapy approach for MPS, suitable for both home and office environments.

**Figure 1:**
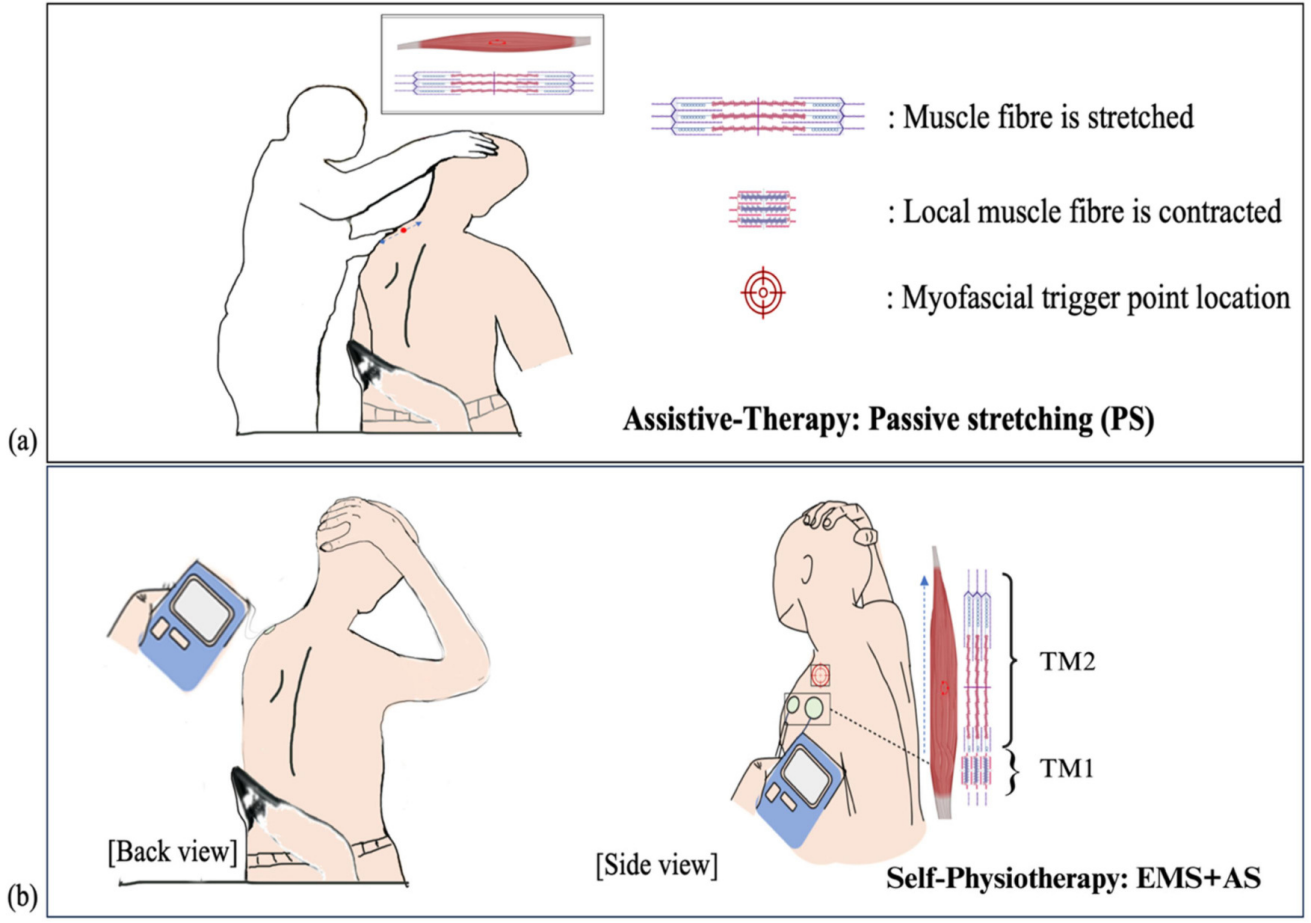
Illustration of passive stretching (PS) therapy, showing the therapist stretching the upper trapezius muscle **(a)** illustration of self-physiotherapy with EMS + aS **(b)** EMS demonstrates localized muscle contraction (black dashed line) between two electrodes, resisting the aS (blue dashed line) that covers the myofascial trigger point (MTrP) location (red spot). TM1 corresponds to the local muscle contraction part, while TM2 corresponds to the stretched part. PS, passive stretching, EMS + AS, electrical muscle stimulation combined with active stretching.

Electrical muscle stimulation combined with active stretching (EMS + AS) is designed to mimic therapist-assisted PS. Both methods are based on the force-length relationship principle, which states that muscle tension decreases as the muscle is elongated (9, 10). Our design showed that EMS indicates a potential pattern of local muscle contraction between two electrodes with a small interelectrode distance (IED), which resisted the AS, and thereby facilitated a greater TM stretch. Therapists typically apply PS by compressing the shoulder blade with one hand while gently stretching the neck in the opposite direction with the other hand (Figure 1A). The effect of EMS is intended to simulate the therapist's hands during PS. For EMS + AS, the local muscle contraction is designated as TM1, and the stretched part as TM2 (Figure 1B). The key to specific local muscle activation is designing two EMS electrode placements that are far from the MTrP and in opposite directions during AS.

This study proposes a new method using EMS for self-physiotherapy. Our previous work has shown that EMS + AS significantly reduces pain compared to sham stimulation (AS alone) and transcutaneous electrical nerve stimulation (TENS) for one minute of treatment (11). However, the effectiveness of EMS + AS has not been validated using ultrasound imaging. We hypothesized that (1) target muscles under EMS + AS can stretch more, and (2) that the treatment performance of EMS + AS on MPS is better than or comparable to PS. To verify these two hypotheses, two experiments were conducted: a preliminary ultrasound-based experiment *(Experiment 1)* designed to verify Hypothesis 1, followed by a comparison study *(Experiment 2)* that utilized measures such as PI, PPT, and sEMG activity to verify Hypothesis 2. MPS is particularly relevant to loss of muscle strength, a factor often disregarded that contributes significantly to MTrP formation and can impair performance (12). Conversely, strengthening the muscles can inhibit MTrP formation (13). Therefore, this study emphasizes the crucial role of assessing participants’ understanding of MPS prevention strategies in enhancing the effectiveness of self-physiotherapy. To further facilitate and inform this move towards self-physiotherapy, additional outcomes, including participants’ feedback, were also analyzed. An effective self-physiotherapy has the potential to empower individuals to proactively slow the progression of MPS, especially given that stresses related to office or home-based work environments can further exacerbate pain severity (14). Self-physiotherapy applied with EMS + AS may offer a convenient option for working adults with MTrPs in the TM.

## 2 Methods

### 2.1 Study design

This study provided all treatments in randomized order (a randomized crossover study). Two experimental studies were conducted at separate visits at the University of Bath laboratory or participants’ accommodations (only Experiment 2). Experiment 1 (preliminary study) involved ultrasound imaging. Experiment 2 (main study) consisted of three steps: 1) collecting personal information, previous MPS treatments, and baseline health status; 2) measuring changes in PI, PPT, and sEMG activity during TM action pre- and post-treatment; and 3) administering feedback questionnaires assessing participants satisfaction, treatment preferences, exercise knowledge for MPS prevention, and qualitative comments. Experiment 1 (4961–6056) and Experiment 2 (EP 22 081) were approved by the University's Biomedical Science Research Ethics Committee. All participants provided written informed consent. All procedures were conducted in accordance with relevant institutional guidelines and regulations, and the study protocol adhered to established ethical standards. Clinical trial registration ID: NCT07315776 (02/01/2026).

#### 2.1.1 Sample size calculation

##### 2.1.1.1 Experiment 1

A systematic review showed the minimum sample size (i.e., eight) for investigating ultrasound imaging on MPS (15). Therefore, a pilot study recruited 8 participants.

##### 2.1.1.2 Experiment 2

A power analysis was conducted to determine the required sample size for pain improvement when comparing stretching for MPS treatments. Based on a systematic review (7), a medium effect size (standardized mean difference of 0.5) was adopted. With an *α* = 0.05 and powe*r* = 0.8 (for a comparative t-test), a minimum of 64 participants was required per group. A total sample size of 65 participants was set for the crossover design.

### 2.2 Participants

**Figure 2:**
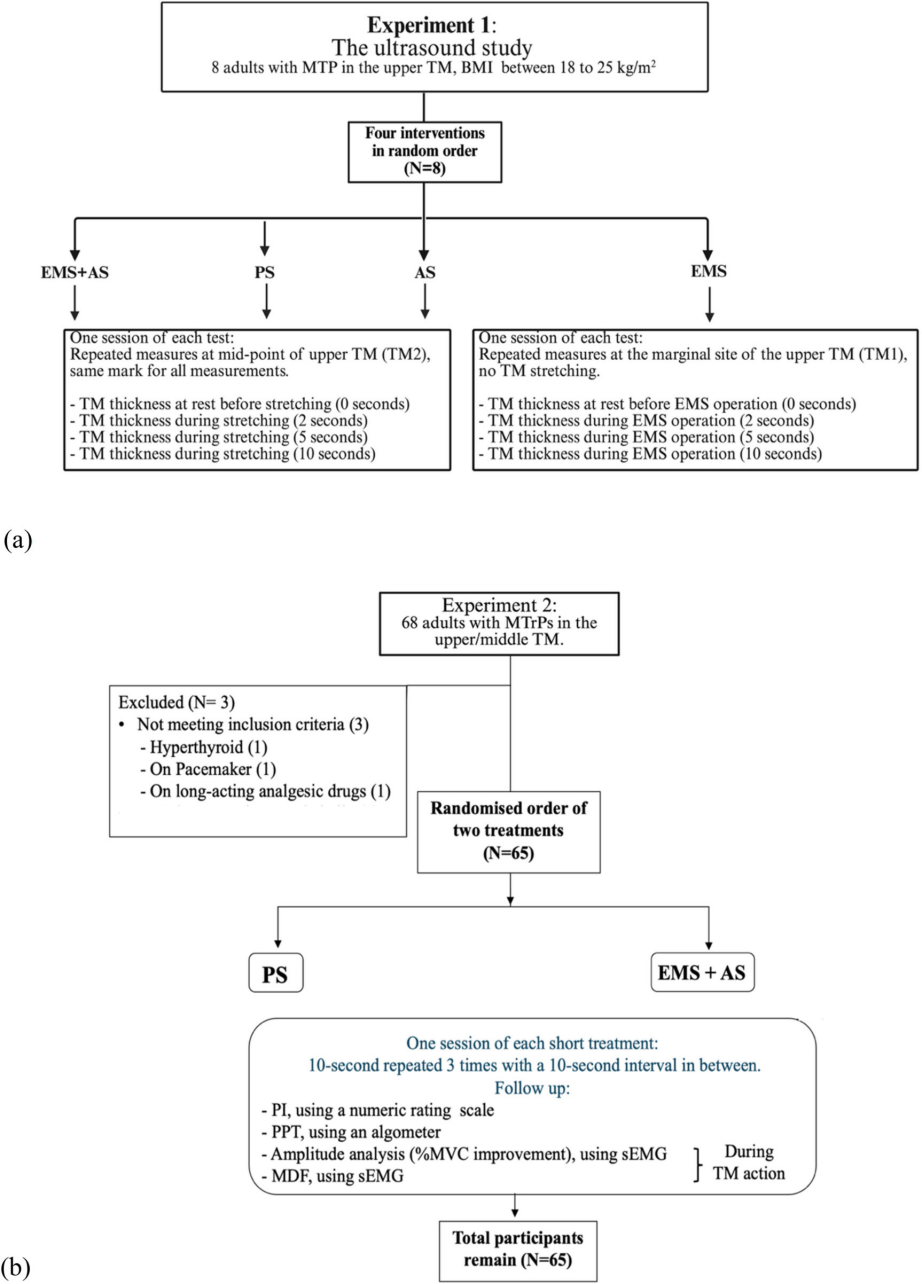
**(A)** Overview of Experiment 1: the ultrasound study in which four interventions—EMS + aS, PS, aS, and EMS—were applied in random order (N = 8). **(b)** Study flow chart for Experiment 2: The main study in which two interventions—EMS + AS and PS—were applied in random order (N = 65).

In both experiments, participants over 18 years old with either active or latent MTrPs in the TM were diagnosed with MPS by a General Practitioner (GP). Inclusion criteria for both experiments required participants to have no history of chronic diseases (cancer, cervical nerve root compression, heart arrhythmias, including hyperthyroid, muscle weakness, seizure, skin infection at the upper back, spinal cord injury, stroke), and have ability to move the upper limbs. Participants also reported no use of pacemakers or strong pain relievers, no allergies to metals, gels, or cleaning products, and no pre-existing severe muscle soreness. Experiment 1 recruited eight participants with normal BMI and MTrPs presented at the upper TM (Figure 2A). Experiment 2 included sixty-five participants with MTrPs in the upper or middle TM, who received randomized PS and EMS + AS treatments. Three of the sixty-eight initial volunteers were excluded for not meeting inclusion criteria (hyperthyroidism, pacemaker, or long-acting analgesics) (Figure 2B).

### 2.3 Interventions

Both experiments involved the PS and EMS + AS treatments. Experiment 1 additionally included AS, where participants pulled their neck away from the MTrP location, and EMS operation at the upper TM margin. Active or latent MTrPs were identified in the upper TM (in both experiments) or the middle TM (in Experiment 2 only). Each treatment consisted of three 10-second stretches with 10-second breaks in prescribed positions. Treatments were administered in a computer-generated randomized order, concealed from participants, with a two-minute break between each treatment. The principal researcher managed the assignment implementation.

**Figure 3:**
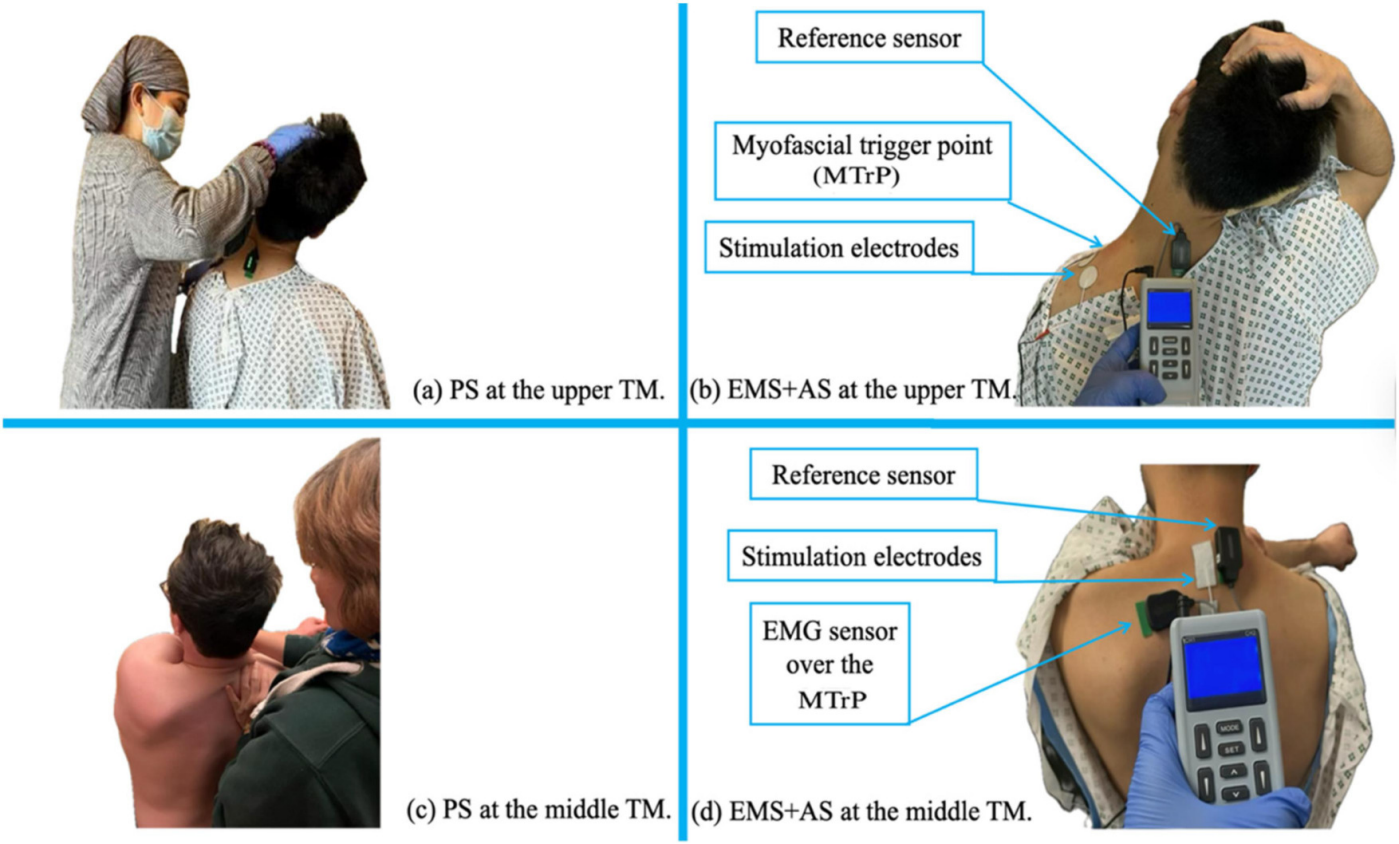
Example of two treatments on the upper **(a,b)** and the middle TM **(c,d)** in this study: for therapist-assisted PS **(a,c)**, and for applying EMS + aS **(b,d)**. EMG, electromyography; EMS + AS, electrical muscle stimulation combined with active stretching; MTrP, myofascial trigger point; PS, passive stretching; TM, trapezius muscle.

For PS, the therapist stretched the upper TM via shoulder depression and neck deviation (Figure 3A). In Experiment 2 only, the middle TM was stretched by scapular protraction while stabilizing the mid-spine and gently pulling the arm inward (Figure 3C).

For EMS + AS, electrical was stimulated using the Med-Fit Digital EMS (EM-6200A) generating an asymmetrical biphasic square waveform (20 Hz frequency, 100 *μ*s pulse width). Amplitude was adjusted for tetanic contraction based on individual tolerance. The protocol targeted superficial muscles (16) by using constant current mode without ramp stimulation and transverse electrodes placement with a small IED across muscle fibers. Two circular electrodes (diameter 32 mm with 1 cm IED) were placed near the acromion at TM1 (Figure 3B). In Experiment 2, two rectangular electrodes (20 × 40 mm with 1 cm IED) were placed on the middle TM near the spine (Figure 3D). EMS electrode placements were positioned in the opposite direction of AS.

### 2.4 Outcome measures

*Experiment 1* involved ultrasound imaging of eight upper TMs. Imaging was performed at the TM2 for three conditions: EMS + AS, PS, and AS alone. Additionally, ultrasound imaging was used at the TM1 during EMS operation. Participants were evaluated for TM thickness at each time point (i.e., 0, 2, 5, and 10 s) during the stretching procedure. The ultrasound acquisition procedure followed a B-mode musculoskeletal ultrasound protocol. A linear transducer GE 9L-D (Verasonics) frequency was set at 7.8 MHz for the superficial muscle layer (a 20–30 mm depth), and ultrasound image settings (time-gain compensation, depth, sector size, etc.) were kept consistent across all participants (17). During image acquisition, participants sat upright with arms relaxed at their sides and hands on their laps. The transducer was oriented for a longitudinal view, positioned perpendicular to the skin, and adjusted to optimize image brightness (18). At TM1 (local muscle contraction), the two EMS electrodes were positioned at the upper TM margin which was marked for transducer placement (19). During EMS operation, the transducer was positioned at this marked location, ensuring no gel-electrode contact. At TM2 (stretched part) during EMS + AS, PS, and AS alone, the transducer was positioned at the TM midpoint, identified as the point between the C7 spinous process and the acromioclavicular joint. Ultrasound gel (2.5 mL) was applied with maintained pressure during contact. The average TM thickness was determined as the distance between the inner border of the superior and the inferior TM sheaths. Measurements were obtained from five vertical lines drawn on the ultrasound images of the upper TM (both TM1 and TM2 regions) and analyzed using the ImageJ software. An excellent intraclass correlation coefficient (ICC = 0.99, 95% CI: 0.99–1.00) was observed for TM thickness measurement by a single rater (a medical doctor), aligning with findings from a previous study (20, 21).

*Experiment 2* involved main and additional outcomes. The main outcomes assessed changes in PI, PPT, MDF, and %MVC during TM action between the PS and EMS + AS treatment groups. Additional outcomes explored the impact of prior MPS treatments on knowledge of preventive exercise methods, as well as participants’ feedback, including qualitative data. For main outcomes, PI was measured using the numeric rating scale (NRS) (0 = no pain, 10 = worst pain), a tool known for its test-retest reliability and validity (22). PPT was assessed using a digital algometer (Wagner, Greenwich, USA). A round rubber tip was used to apply constant pressure until the participant reported the first onset of pain, with the force recorded in kg/cm^2^ (kgf) (23). sEMG activity was recorded using a 16-channel sEMG (Delsys Trigno Maize Sensor system). Each sensor featured 2 mm diameter silver contacts (6 mm IED); the rectangular 5 × 1 mm 4-channel reference sensor was placed on the C7 spinous process (24) After skin abrasion with crystal pads and alcohol cleaning, sensors were positioned on the MTrP, commonly located on the muscle belly. Data acquisition involved recording three sets of maximum TM actions. sEMG was measured during shoulder elevation (upper TM) and scapular retraction (middle TM) pre- and post-treatment using Trigno Discover (v1.6.5). 16 channels with a good signal-to-noise ratio (SNR) were selected for precise EMG data. Three peak sections from the highest three actions within defined time windows were identified using a self-developed MATLAB script (MATLAB software R2024b), and the median frequency (MDF) was calculated for each. For amplitude analysis, the time-amplitude integral of the root mean square (RMS) quantified EMG data per channel. To minimize the influence of regional anatomical differences—even though measurements were obtained during different actions, post-treatment RMS amplitude values were normalized to pre-treatment values using EMG Works analysis software version 4, to determine the mean percentage of maximum voluntary contraction (%MVC) improvement.

### 2.5 Statistical analysis

*For both experiments,* mean and standard deviation (SD) were calculated, including overall mean difference (MD) values and standard error (S.E.) at pre- and post-treatment. A significance level of 0.05 was used for all analyses, with 95% confidence intervals reported. In Experiment 1, as the data were normally distributed, paired samples -tests were used to compare MD values. thickness MD values at each time point were compared between EMS + AS and PS, and between EMS + AS and AS alone. Repeated-measures ANOVA was conducted across the three different time points over the 10-second interval. In Experiment 2, as the data for changes in PI, PPT, MDF, and %MVC were not normally distributed, Mann–Whitney U tests were used to compare median and mean rank values between the two groups. Data were analyzed using SPSS software (version 31). Percent differences between the two treatments were calculated to determine degree of improvement when an outcome measure showed a significant difference.

*For additional outcomes of Experiment 2*, three analyses were conducted. First, descriptive data from the health survey, administered prior to the experiment (included upper back muscle stretching), and past MPS treatments received were reported. Second, a binary logistic regression (backward stepwise conditional method) was used to determine the effects of the most frequently utilized self-care approaches for MPS, identified from the survey, on participants’ knowledge of MPS prevention exercise methods. This model showed good fit to the data, with the dependent variable coded as 1 for knowledge of both strengthening and stretching exercises, and 0 for knowledge of either. Third, to report percentages of participants’ thoughts on treatment effectiveness and satisfaction, a paired t-test on scores was conducted to determine significant differences between the two treatments. Additionally, qualitative data from participants’ feedback were organized into three structural domains: overall impression, lack of satisfaction, and suggestions for improvement, which emerged directly from a careful review and categorization of recurring themes within the participants’ verbatim comments. The principal researcher collected all anonymous data for blinded analysis. Missing data were handled by exclusion (complete case analysis), which was based on quality control criteria, such as selecting data with an adequate SNR.

## 3 Results

The two experimental studies recorded participants’ characteristics and baseline data (Table 1). No participant withdrawals, adverse events, or use of concomitant care were reported. Details of occupations among working adults included business workers (*n* = 10), kitchen workers (*n* = 8), hospital workers (*n* = 6), computer-based workers (*n* = 5), nursery workers (*n* = 4), and academic staff (*n* = 3).

**Table 1:** Participant characteristics by experiment.

Participant characteristics	Experiment 1 (*n* = 8)	Experiment 2 (*n* = 65)
Age (years old)	29.75 ± 3.24	37.47 ± 12.51
BMI (kg/m^2^)	22.87 ± 2.07	24.07 ± 4.89
Sex (F/M)	4F/4M	41F/24M
Ethnicity (Asian/non-Asian)	8/0	43/22
Occupations (Students/Working adults)	8/0	29/36
Active/Latent MTrPs	2/6	15/50
Number of Interventions	4 (AS, PS, EMS + AS, and EMS)	2 (PS, and EMS + AS)

Data are presented as mean ± standard deviation, or number of participants, Students means postgraduate students.

AS, active stretching; BMI, body mass index; EMS, electrical muscle stimulation operation; EMS + AS, electrical muscle stimulation combined with active stretching; F, females; M, males; MTrPs, myofascial trigger points; PS, passive stretching.

### 3.1 Primary outcomes

#### 3.1.1 Experiment 1

**Figure 4:**
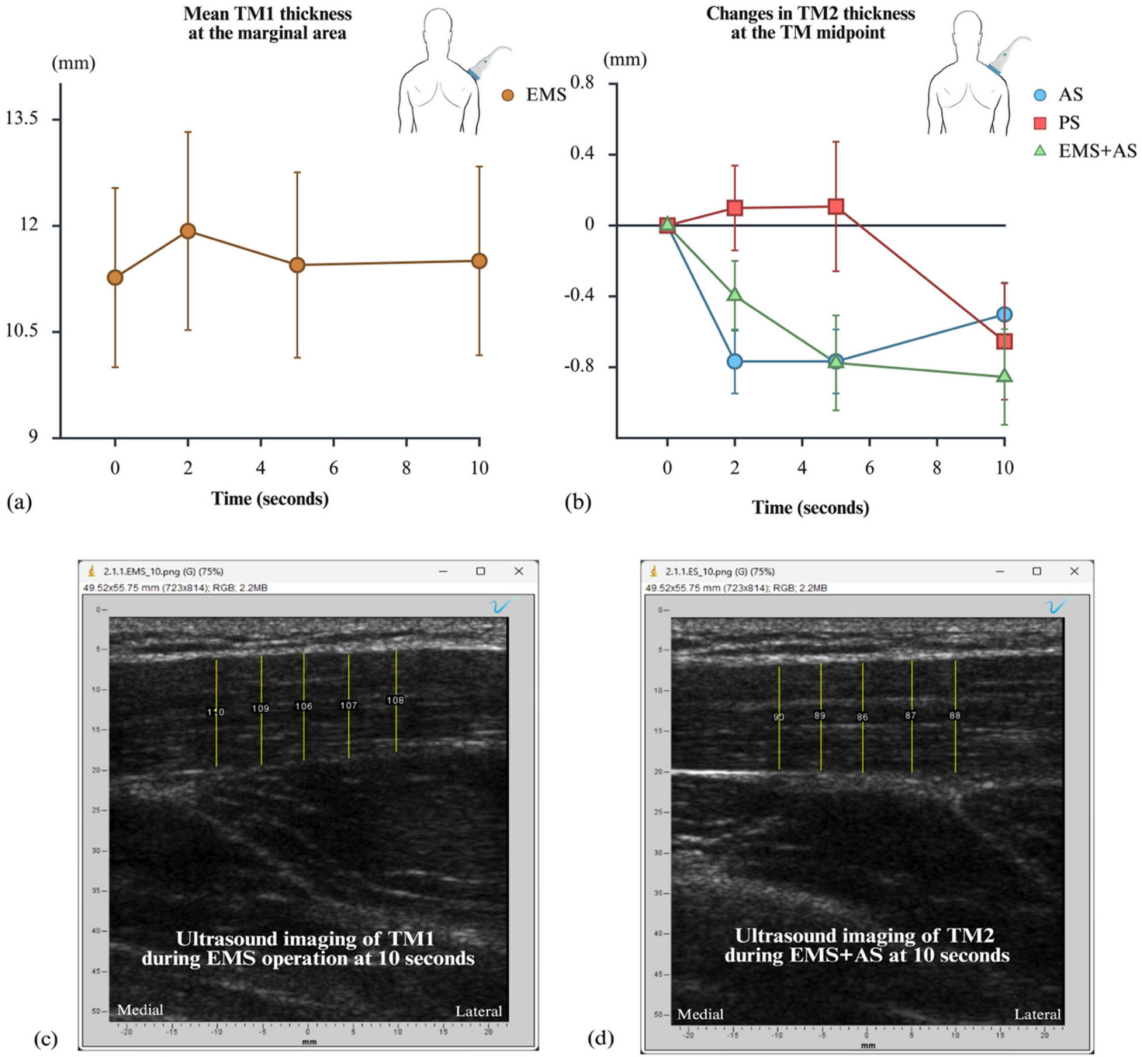
Illustration of TM1 thickness at 2-, 5-, and 10-seconds during EMS operation at the margin of the upper TM **(a)** illustration of changes in TM2 thickness at 2-, 5-, and 10-seconds during EMS + aS (green line), PS (red line), and aS (blue line) treatments **(b)** the transducer locations are shown above the graphs, and the error bars represent standard error. Examples of ultrasound imaging are shown for TM1 during EMS operation **(c)** and for TM2 during EMS + AS **(d)**, both taken at 10 s (five vertical lines were averaged to determine TM thickness). AS, active stretching; EMS, electrical muscle stimulation; EMS + AS, electrical muscle stimulation combined with active stretching; PS, passive stretching; TM, trapezius muscle; TM1, trapezius muscle at local contraction part; TM2, trapezius muscle at stretched part.

TM thickness was observed at TM1 during EMS operation and at TM2 during EMS + AS, PS, and AS. All measurements were taken at 2, 5, and 10 s. At TM1, EMS was applied at 17.38 ± 6.23 mA during operation; the baseline TM1 thickness was 11.27 ± 3.58 mm. The thickness then increased to 11.93 ± 3.96 mm at 2 s, 11.45 ± 3.70 mm at 5 s, and 11.51 ± 3.77 mm at 10 s, with no significant difference between time points (*p* > 0.05). At TM2, EMS + AS provided constant resistance, resulting in a consistent reduction in TM thickness over 10 s compared to TM thickness at rest. However, TM2 thickness hints at a preliminary trend of inconsistent decline over 10 s during PS and AS alone. Figure 4 shows the mean TM thickness measured at TM1 during EMS operation (Figure 4A) and the changes in TM thickness at TM2 during EMS + AS, PS, and AS at each time point (Figure 4B). At 10 s, examples of ultrasound imaging are displayed for TM1 thickness during EMS operation (Figure 4C) and for TM2 thickness during EMS + AS (Figure 4D). No significant differences were observed between EMS + AS and PS or between EMS + AS and AS alone (*p* > 0.05) (Table 2). Comparative evaluation of changes in TM thickness at the stretched region showed no significant differences across time points (*p* > 0.05); AS: F_(2,14)_ = 0.598, *p* = 0.564, PS: F_(2,14)_ = 3.092, *p* = 0.077, EMS + AS: F_(2,14)_ = 1.709, *p* = 0.217.

**Table 2:** The mean differences and standard error (S.E.), and *t*-value (95% CI) for changes in TM thickness at TM2 (EMS + AS vs. PS; EMS + AS vs. AS).

	Changes in TM thickness (mm)			Mean Difference (S.E.) (mm)		t value (95%CI)	
**Time (s)**	EMS + AS	PS	AS	EMS + AS and PS	EMS + AS and AS	EMS + AS and PS	EMS + AS and AS
2	−0.40	0.10	−0.77	−0.50 (0.29)	0.37 (0.29)	−1.70 (−1.19, 0.19)	1.27 (−0.32, 1.06)
5	−0.78	0.11	−0.65	−0.88 (0.38)	−0.12 (0.31)	−2.34 (−1.78, 0.01)	−0.39 (−0.87, 0.62)
10	−0.86	−0.65	−0.50	−0.20 (0.20)	−0.35 (0.23)	−0.98 (−0.68, 0.28)	−1.53 (−0.90, 0.19)

AS, active stretching; CI, confidence interval; EMS + AS, electrical muscle stimulation combined with active stretching; PS, passive stretching; s, seconds; S.E. = standard error; TM, trapezius muscle; TM2, trapezius muscle at stretched part.

#### 3.1.2 Experiment 2

**Figure 5:**
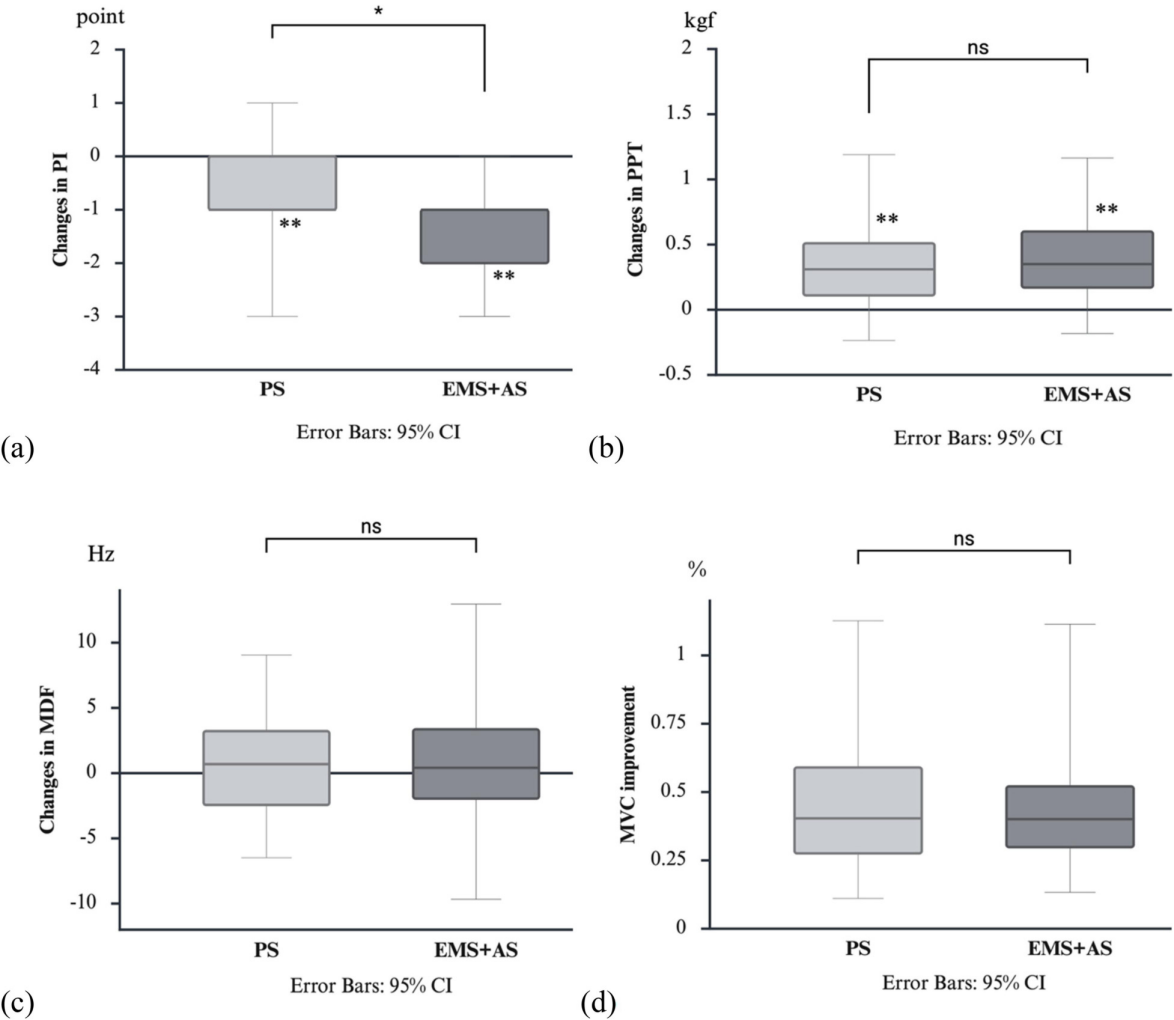
Illustration of changes in PI **(a)**, PPT **(b)**, MDF **(c)**, and %MVC improvement **(d)** before and after PS and EMS + aS treatments. **p < 0.001 denotes a significant change from baseline within a treatment. *The black line indicates a significant difference between the two treatments (*p* < 0.05), while ns indicates no significant difference. EMS + AS, electrical muscle stimulation combined with active stretching; kgf, kilogram force (kg/cm^2^); MDF, median frequency; MVC, muscle voluntary contraction; ns, non-significant; PI, pain intensity; PPT, pressure pain threshold; PS, passive stretching.

Participants reported an overall moderate pain level for MPS (6.00 ± 1.35) prior to the study. Mean duration since GP notification was 2.40 ± 3.76 years; mean symptom duration was 2.17 ± 3.38 years. Before the study, participants’ most common self-care approaches for MPS included: stretching, massage, exercises, ergonomic adjustment, external balm, analgesic drugs, yoga, warm compression, self-myofascial release, and meditation. While 73.8% performed body stretching, only 46.2% performed TM stretches (shoulder depression and scapular retraction). Additionally, 56.9% engaged in exercises, primarily aerobic and resistance training. Post-treatment EMS amplitude was 19.62 ± 11.99 mA. Both treatments significantly reduced PI and increased PPT, *p* < 0.001. Changes in PI were significantly greater in the EMS + AS group (Mean Rank=59.22, −1.15 ± 0.13) than in the PS group (Mean Rank=71.78, −0.77 ± 0.15), U = 1,704.50, *p* = 0.047. A mean rank difference of 17.50% was observed between the two treatments. However, the changes in PPT (Mean Rank of EMS + AS = 68.25, Mean Rank of PS = 62.75; U = 1,933.50, *p* = 0.405), changes in MDF (Mean Rank of EMS + AS = 65.23, Mean Rank of PS = 65.77; U = 2,095.00, *p* = 0.935), and %MVC improvement (Mean Rank of EMS + AS = 66.06, Mean Rank of PS = 64.94; U = 2,076.00, *p* = 0.865) were not statistically significant (Figure 5).

### 3.2 Additional outcomes

Among respondents, 63.1% acknowledged the benefits of both TM stretching and strengthening exercises, while 32.3% were unaware that strengthening exercises can reduce pain. A binary logistic regression assessed the influence of the most common self-care MPS treatments on participants’ knowledge of MPS prevention exercise methods. Only stretching experience was a significant predictor: participants with prior stretching experience were 5.11 times more likely to correctly identify both exercise types as beneficial [OR = 5.11, 95% CI (1.40; 18.70), *p* = 0.014]. This is further supported by the 48 participants (73.8%) with stretching experience who correctly identified the combined benefits of both strengthening and stretching exercises for MPS prevention.

**Figure 6:**
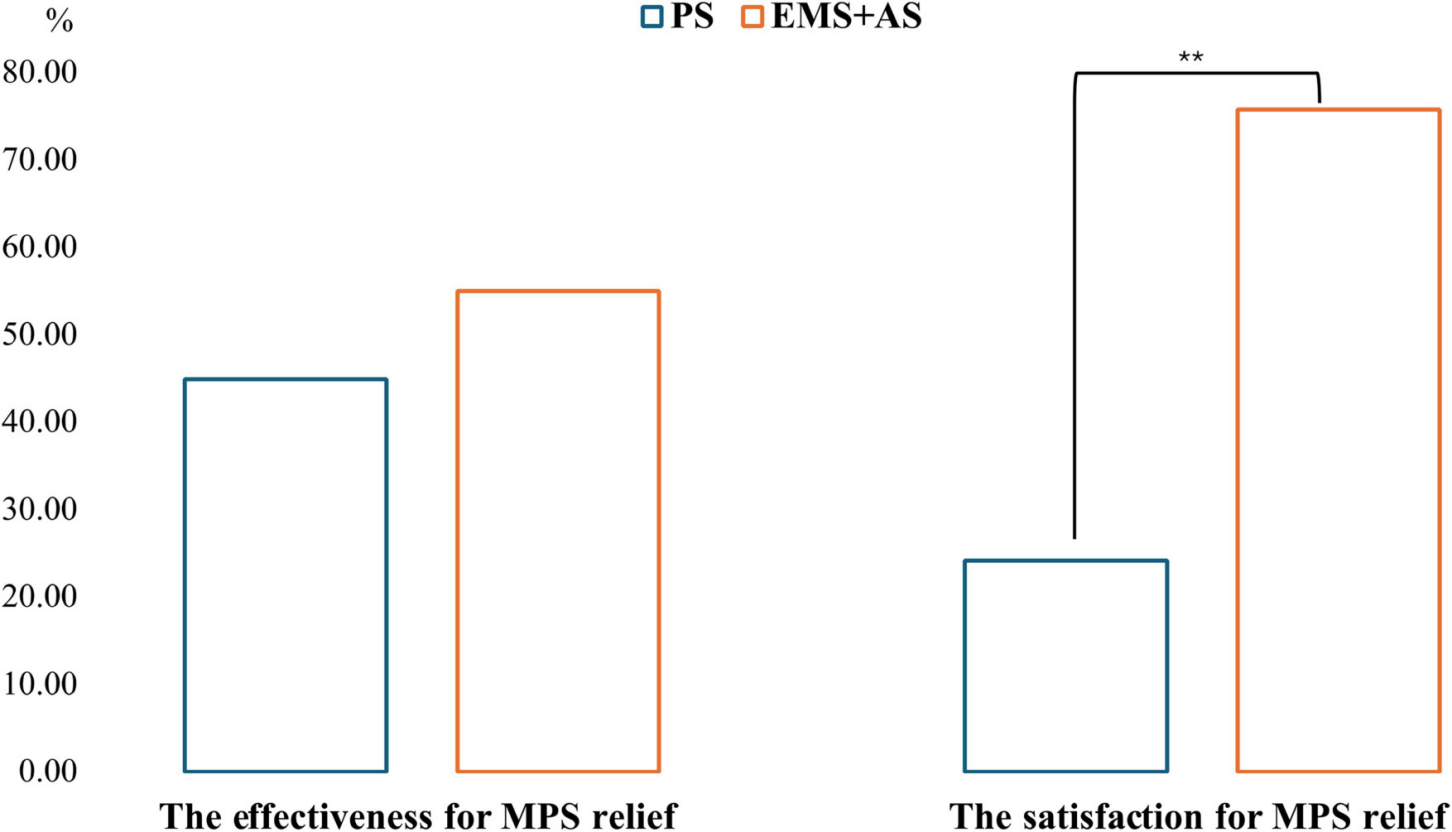
Illustration of participant feedback on the most effective and satisfying treatment for myofascial pain relief. **p < 0.01: significant difference between the two treatments.

Participants rated EMS + AS higher in both effectiveness (55.00%) and satisfaction (75.70%) for MPS treatment compared to PS (45.00% and 24.30%, respectively). Only the satisfaction score of EMS + AS significantly increased compared to PS, t_(64)_ = 3.15, 95% CI [0.10; 0.43], *p* = 0.002) (Figure 6). While most participants (72.30%) believed EMS enabled TM stretching, 10% were uncertain about its effect, and the rest thought EMS did not help to stretch.

Qualitative data reflected participants’ varying perspectives on the two treatments: EMS + AS and therapist-assisted PS.

- Overall Impression: Participants generally reported EMS + AS as more effective and relaxing post-stimulation. The EMS was surprisingly appreciated for its role in assisting the stretch. PS was valued specifically for allowing the neck to be stretched beyond self-imposed limits and was linked to pain relief.
- Lack of Satisfaction: Feedback for EMS + AS was mixed, with some participants expressing uncertainty about the stretching sensation. In contrast, PS received negative feedback, with some finding it boring and others reporting discomfort.
- Suggestions: The EMS + AS treatment was viewed as having potential to benefit a large number of individuals. Despite the mixed reviews for PS, participants recognized that regular stretching remains crucial for long-term pain management.

## 4 Discussion

Both groups (Experiment 1 and Experiment 2) experience similar sustained loading of TM, whether from prolonged sitting or ergonomic work demands. This static load is the primary mechanical contributor to MTrP formation, and the underlying energy-crisis mechanism is consistent across individuals and anatomical regions, even though the upper and middle TM groups were analyzed together rather than separately. However, our analysis focused on within-subject changes in PI, PPT, and EMG activity in Experiment 2, and changes in TM thickness in Experiment 1 (the pilot ultrasound study). Therefore, demographic differences between the two experiments (e.g., age and occupation) did not influence the outcomes, as each experiment assessed different outcome measures. Experiment 1 provides hypothesis-generating rather than confirmatory evidence. The primary intent in Experiment 2 was to evaluate clinical and electrophysiological outcomes independently of the inconclusive mechanistic trend observed in Experiment 1. A key design limitation of Experiment 2 is the lack of an AS-only control group. Because EMS was delivered concurrently with AS (EMS + AS), any observed improvements may reflect the mechanical effects of stretching, the electrical stimulation, or their combination. Future studies should therefore incorporate an AS-alone arm, and ideally a sham-EMS condition to quantify the incremental contribution of electrical stimulation beyond mechanical stretch and to clarify the specific mechanism of action. The following discussion therefore considers the results of Experiments 1 and 2 separately but in parallel, acknowledging the exploratory nature of the ultrasound findings and the confirmatory clinical outcomes.

Experiment 1 suggests a possible trend that changes in TM thickness related to the stretch interventions: a decrease in TM2 thickness implied greater stretching, which was associated with pain reduction observed in Experiment 2. Conversely, an increase in TM1 thickness implied muscle shortening, which contributed to resistance at TM2. In the first part, EMS-generated muscle contraction caused TM1 shortening, which showed a maximum increase at 2 s, followed by a slight decline at 5 and 10 s. Despite this decline, the TM1 thickness at the margin of the upper TM remained above the baseline measured at TM rest. This result aligns with previous findings that TM thickness increases during muscle contraction (21). This finding exhibits a suggestive pattern consistent with our hypothesis that TM1 assisted the stretched part (TM2). The consistent decrease in TM2 thickness during muscle stretching with EMS + AS suggests an improvement in muscle tone (20). and implies greater stretching of the upper TM. This indirect inference is necessary because direct measurement of the stretching length along the longitudinal line of the TM is challenging due to the muscle's long, fusiform fiber arrangement (17). Although EMS + AS produced a slightly superior effect compared to PS and AS alone, there was no statistically significant difference. During AS, TM thickness decreased before gradually increasing towards the end, possibly due to individual effort or inconsistent pulling force. In contrast, PS caused an initial increase in TM thickness, possibly due to insufficient muscle relaxation from rapid changes in muscle length (where elevated muscle tone, triggered by a stretch reflex, excited muscle activity, leading to increased TM thickness) (20) or discomfort from the therapist's maximum assisting force (10). TM thickness then decreased as the end of the phase. For EMS + AS, TM thickness gradually decreased throughout stretching, which may be attributed to the sustained resistive force generated by the EMS. This sustained resistance helps break adhesions around MTrPs and reduces acetylcholine (ACh) levels. ACh is the neurotransmitter responsible for muscle contraction (25). Specifically, from participants’ feedback, muscle relaxation observed after EMS cessation likely stems from the gradual release of tension in muscle fibers, returning to rest. This effect is further influenced by the sympathetic modulation and endorphin release induced during and after the electrical stimulation (26). EMS allows targeted muscle contraction with minimal antagonistic interference, providing greater comfort, whereas therapist-assisted PS has also been associated with some reported discomfort in prior research (9, 10). This directly contributed to the significantly higher satisfaction observed with EMS + AS. Participants’ positive experience aligns with objective treatment efficacy findings, as ultrasound imaging consistently showed TM thickness reduction (Figures 4b,d).

Experiment 2 included main and additional outcomes (participant knowledge and feedback) for the stretch experiment. Although both EMS + AS and PS showed significant improvements in PI and PPT between before and after treatments, EMS + AS shows an emerging pattern of significantly greater reduction in PI compared to PS. Although the 17.50% difference in mean rank is not an intuitive measure for clinical interpretation, the mean pain reduction of 1.15 points on the 11-point NRS scale represents a modest change, as the minimally clinically important difference (MCID) for musculoskeletal pain typically ranges between 1.3–1.5 points (27, 28). Therefore, our observed reduction approaches, but does not fully reach, the lower threshold of the MCID. This is caused by EMS activating the muscle spindles and the Golgi tendon organ (located near the acromion or spine) (26), facilitating slight muscle elongation and alleviating muscle fiber tension after treatment (3, 10, 29). This highlights a key advantage of EMS + AS in pain reduction. Additionally, changes in PPT, MDF, and %MVC suggest EMS + AS effects are similar to PS. Both treatments prevent prolonged muscle activation and mitigating pain, thereby improving PPT (13, 25, 30), consequently enhancing more efficient TM effort. Increased sEMG activity during TM action aligns with previous finding showing higher sEMG in non-MTrP regions during TM function (6), often attributed to sympathetic facilitation (29). The acute sEMG changes during TM action observe in this study may serve as indicator of MTrP pathophysiology changes, as a previous study revealed that changes in MDF were correlated with the quantity of PPT in the MTrPs (6). In summary, EMS + AS suggests a possible trend of greater benefit than PS for pain reduction but showed similar effects on PPT and sEMG activity.

## 5 Strengths and limitations

Experiment 1 was a pilot study, to confirm the acute effect of EMS + AS and hinted at a preliminary trend of TM thickness increases at TM1 and consistent decreases at TM2. Although this trend alone is insufficient to confirm that EMS + AS produced greater stretching, the exploratory purpose of Experiment 1 was to observe potential physiological tendencies that may reflect measurement variability inherent to ultrasound imaging. Future studies with larger sample sizes and a dedicated protocol for assessing MTrPs are needed to validate the hypothesized mechanism. However, ultrasound measurements further require multiple clinicians to improve observational accuracy. For the main outcomes, the 17.50% between-group difference in pain reduction, accompanied by the borderline *p*-value (*p* = 0.047), shows that the mean reduction of 1.15 points on the 11-point NRS represents only a modest change, as the MCID for musculoskeletal pain typically ranges from 1.3 to 1.5 points (27, 28). This indicates a statistically detectable but modest effect, though its robustness is limited. Experiment 2 lacked a control group for comparison, such as AS, which may have limited the detection of treatment differences. The timing of EMG measurement is critical to account for transient effects. Previous studies have reported that after massage, muscle contraction time rapidly returns to baseline (31), while EMG activity declines more slowly and typically remains above baseline for only 2 min (32). Therefore, selecting a 2-minute interval for post-treatment measurement provides an adequate washout period for obtaining meaningful EMG data in MPS research. This study suggests a possible trend that EMS can complement TM stretching, functioning in a similar manner to PS and thereby challenging the application of a minimum treatment duration in line with practical recommendations on stretching exercises (10). EMS + AS shows promise as a self-physiotherapy for MPS and indicates a potential pattern of significantly superior pain reduction and satisfaction over PS. Strong muscles promote greater energy and help prevent the loading of muscles to a critical point of chronic MPS development (13). Therefore, providing accurate knowledge about integrating strengthening with stretching is crucial for effective self-physiotherapy in MPS rehabilitation and prevention of MTrPs.

To promote exercise self-efficacy for chronic muscle pain management, thus, exploring participants’ knowledge and feedback proves worthwhile (33). As shown in our research, participants perceived stretching alone as insufficient for MPS rehabilitation because pain persisted, leading individuals to recognize the need for strengthening exercises in a combined approach (13). EMS fulfils the requirements of self-physiotherapy and has promising potential for implementation with working adults, particularly when integrated with ergonomic and workplace adjustments to prevent the risk of MPS (34). However, stretch had a smaller effect size on PI and PPT, compared to massage (7), which trigger point pressure release (TPR) can change in biochemical aspects (e.g., glucose and lactate) (35). Therefore, EMS + AS with TPR will be further investigated, as combination MPS therapy has been recommended for greater pain improvements (36).

## 6 Conclusion

Working adults require a tool that extends beyond simple stretching to promote lasting rehabilitation. EMS can bridge the self-physiotherapy gap for managing MPS. The pilot study identified exploratory trends suggesting potential physiological effects, while the main study suggests a possible trend that EMS + AS may serve as a beneficial adjunct for MPS management. Overall, the findings reveal a tentative trend of short-term pain reduction with EMS + AS, but do not demonstrate consistent superiority across all outcome measures. Specifically, EMS + AS provided short-term subjective pain relief and greater user acceptability, whereas objective neuromuscular outcomes were comparable between EMS + AS and PS. Although a one-minute intervention is unlikely to produce structural or morphological changes in MTrP pathology, as snapping nodules within the MTrP remained palpable, the one-minute treatment duration was intentionally chosen based on the proof-of-concept and feasibility-focused aims of this study. This time-efficient protocol reflects real-world self-care conditions, associating the translational relevance of EMS + AS as an accessible adjunct to self-physiotherapy. Future randomized trials with sham stimulation (AS-only control) should examine longer treatment durations and repeated sessions to determine whether sustained EMS + AS can induce clinically meaningful structural changes in MTrPs during a full-course protocol (2–7 rounds) that mimics PS. This approach may greatly improve sEMG activity outcomes. The proposed EMS system may be further developed as a programmable shoulder-pad device (e.g., triggered every 20 min) to assist self-physiotherapy during AS and promote greater muscle elongation.

## Data Availability

The raw data supporting the conclusions of this article will be made available by the authors, without undue reservation.
